# Postoperative Nausea and Vomiting in Female Patients Undergoing Breast and Gynecological Surgery: A Narrative Review of Risk Factors and Prophylaxis

**DOI:** 10.3389/fmed.2022.909982

**Published:** 2022-07-01

**Authors:** Marco Echeverria-Villalobos, Juan Fiorda-Diaz, Alberto Uribe, Sergio D. Bergese

**Affiliations:** ^1^Department of Anesthesiology, The Ohio State University Wexner Medical Center, Columbus, OH, United States; ^2^Department of Anesthesiology, Health Sciences Center, School of Medicine, Stony Brook University, New York, NY, United States

**Keywords:** postoperative nausea and vomiting, female gender, gynecological surgery, breast surgery, randomized clinical trial ERAS (Enhance Recovery After Surgery)

## Abstract

Postoperative nausea and vomiting (PONV) have been widely studied as a multifactorial entity, being of female gender the strongest risk factor. Reported PONV incidence in female surgical populations is extremely variable among randomized clinical trials. In this narrative review, we intend to summarize the incidence, independent predictors, pharmacological and non-pharmacological interventions for PONV reported in recently published clinical trials carried out in female patients undergoing breast and gynecologic surgery, as well as the implications of the anesthetic agents on the incidence of PONV. A literature search of manuscripts describing PONV management in female surgical populations (breast surgery and gynecologic surgery) was carried out in PubMed, MEDLINE, and Embase databases. Postoperative nausea and vomiting incidence were highly variable in patients receiving placebo or no prophylaxis among RCTs whereas consistent results were observed in patients receiving 1 or 2 prophylactic interventions for PONV. Despite efforts made, a considerable number of female patients still experienced significant PONV. It is critical for the anesthesia provider to be aware that the coexistence of independent risk factors such as the level of sex hormones (pre- and postmenopausal), preoperative anxiety or depression, pharmacogenomic pleomorphisms, and ethnicity further enhances the probability of experiencing PONV in female patients. Future RCTs should closely assess the overall risk of PONV in female patients considering patient- and surgery-related factors, and the level of compliance with current guidelines for prevention and management of PONV.

## Introduction

Postoperative nausea and vomiting (PONV) are one of the main distressing symptoms commonly reported after surgery and prompt patients at risk to serious complications, such as gastric aspiration, psychological distress, wound dehiscence, deferred recovery, and prolonged discharge times. Female gender is considered an independent predictor of PONV, being a determinant factor when assessing its preoperative risk (1–3). The Society for Ambulatory Anesthesia (SAMBA) Guidelines for PONV management recommend a multimodal approach or combination therapy consisting of two or more interventions in patients with moderate and high risk of PONV, respectively (4, 5). Although the pathophysiology of PONV is multifactorial, PONV is more insidious in female surgical patients than in male, including elderly patients (6). Women also show a higher susceptibility to motion sickness during air, water, and terrestrial travel, which further increases their risk of PONV (1–3). Several studies have demonstrated an association between hormonal changes and PONV in females at a reproductive age (7–10). Nevertheless, current reports on the frequency of PONV during pre-ovulatory (proliferative) and post-ovulatory (luteal) phases of the menstrual cycle are controversial (7, 9–11).

Current literature describing PONV in female patients undergoing breast and/or gynecological surgery is highly variable in terms of incidence, predictors, risk stratification and management. Several reviews, protocols and guidelines have attempted to summarize PONV management in the general population,. We reviewed the most recent evidence on the impact of PONV occurrence after breast and gynecological surgery to summarize the reported specific considerations about the incidence, independent predictors, and perioperative management (pharmacological and non-pharmacological). Furthermore, we consider that this extensive review of the literature that we have carried out can provide us with a more precise view of some aspects of the clinical spectrum of PONV in female surgical patients that should require a systematic review and meta-analysis.

### Objectives of the Review

To determine the incidence of PONV in female patients undergoing breast and/ or gynecological surgery.

- To identify independent predictors and risk factors for PONV in this subset of patients, although they apply to the female surgical population
- To evaluate the pharmacological and non-pharmacological strategies most currently used for the prophylactic and therapeutic management of PONV.
- To assess the influence of anesthetic agents on PONV occurrence and clarify the optimal anesthetic technique.
- To evaluate the efficacy of the most widely used risk-scoring systems in the risk stratification for PONV in the female surgical population.

### Aims

To Provide updated knowledge to anesthesia providers about key elements that allow them to optimize the perioperative management of PONV in the female population.

## Methods

The research question was formulated according to the PICO methodology. P = Women undergoing breast or gynecological surgery; I = Prevention and treatment of PONV; C = Premenopausal and postmenopausal adult female surgical patients; O = independent predictors and risk factors, risk stratification, available therapeutic strategies, anesthetic management in high-risk patients for PONV.

**Figure 1:**
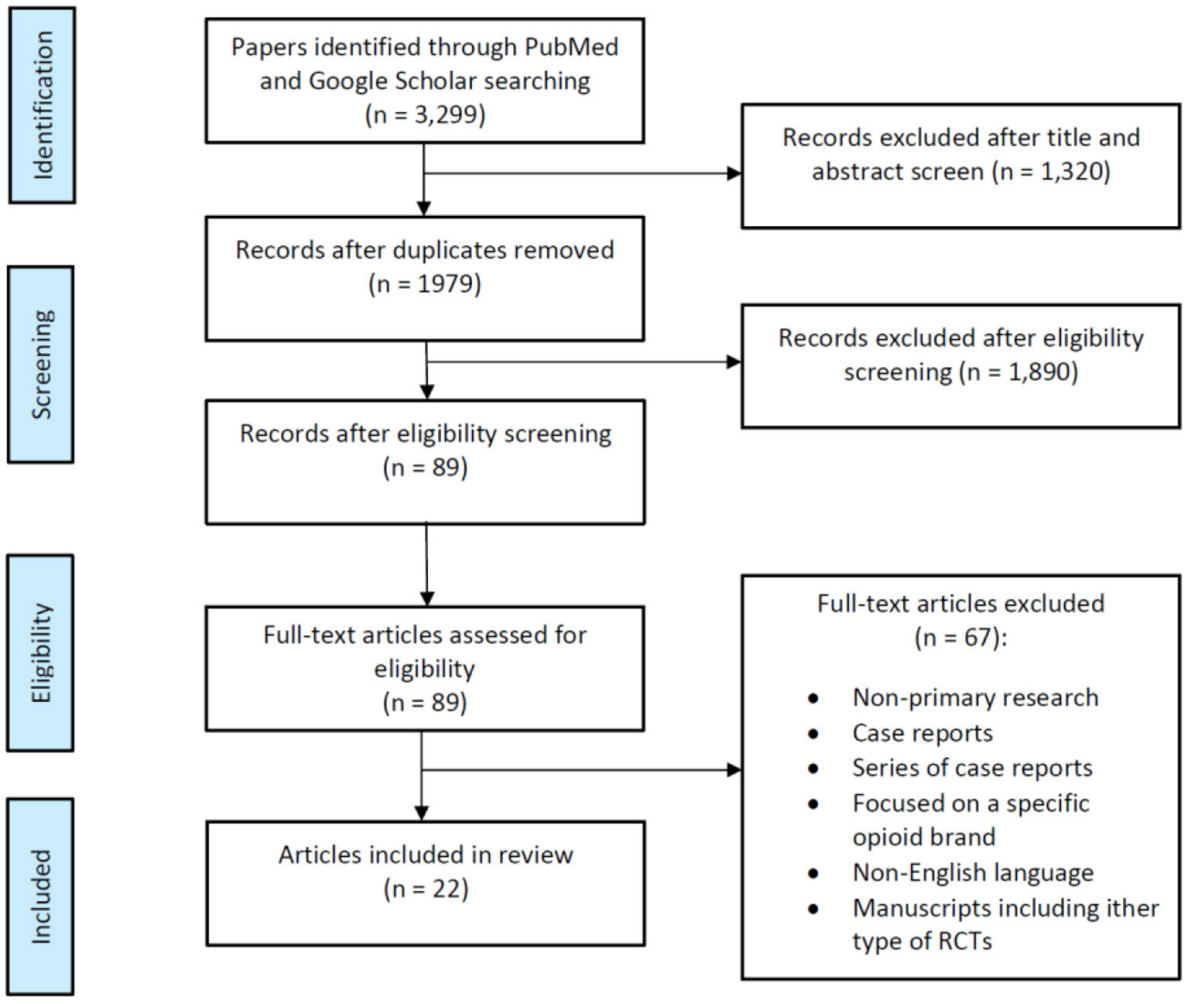
Flow diagram summarizing the selection of randomized clinical trials (RCTs) describing postoperative nausea and vomiting (PONV) in female surgical population.

We performed an extensive literature search in PubMed, MEDLINE, and Embase databases of articles describing PONV management in female surgical populations (i.e., breast surgery and gynecologic surgery) published between January 1, 2011, and June 30, 2021, following the Preferred Reporting Items for Systematic Reviews and meta-Analysis (PRISMA) guidelines (Figure 1) (12). Initially, we use the following keywords and Medical Subject Headings (MeSH) terms: “postoperative nausea and vomiting,” “PONV,” “female gender,” “gynecological surgery,” “breast surgery” and their combinations were used. Thereafter, the following systematic search strategy was used: (PONV OR postoperative nausea and vomiting OR nausea and vomiting, postoperative OR postoperative vomiting OR vomiting, postoperative OR nausea, postoperative OR emesis, postoperative, postoperative, OR postoperative emesis OR postoperative nausea OR antiemetic effect OR complete response) AND (gynecological procedure OR gynecological surgery OR breast surgery OR mastectomy OR mammaplasty) AND (female OR woman). With the results of the initial electronic search, two authors hand-screened several to confirm the following eligibility criteria: Articles published in English language between January 1, 2011, and June 30, 2021, reporting PONV as a primary outcome and describing PONV management in female patients undergoing either breast or gynecologic surgery were included. In addition, our literature search included retrospective studies, systematic reviews, meta-analyses, and review articles from a cited reference search. We excluded conference abstracts and posters, reviews of non-primary research, case reports, series of case reports and articles published in other language other than English. All authors conducted the final review of all databases in July 2021.

## Results

Our database search identified a total of 3,299 articles. After 1,320 duplicated articles were removed, 1,979 articles underwent title and abstract screening. Following this, we selected around 89 publications as reliable articles addressing exclusively PONV in females and screened for eligibility (Figure 1). Among these 89 articles, 67 were excluded for various reasons as shown in Figure 1, and we finally identified a total of 22 eligible publications with a significant number of patients and relevant compilation of demographic and clinical outcomes (Table 1). This is a narrative review; therefore no statistical analysis was performed.

**Table 1:** Randomized clinical trials and postoperative nausea and vomiting outcomes.

**References**	**Surgery type**	**Anesthesia type**	**N/Groups**	**Dose active/Control**	**PONV incidence**
D'souza et al. (13)	Lap. Gyn	Inhaled	31/31/31	Dexamethasone 4 mg / dexamethasone 8 mg / ondansetron 4 mg	29% / 43% / 61% of PONV at 24 h, *p = 0.16*
Ekinci et al. (14)	Lap/open Gyn	Inhaled	20/20/20/20 /20	Droperidol 2.5 mg / metoclopramide 10 mg / tropisetron 2.5 mg / ondansetron 4 mg / control	20% / 40% / 25% / 15% / 60% at 24 h; drop. vs. control *p < 0.009*; Trop. vs. control *p < 0.02*; Ond. vs. control *p < 0.003*
Park and Cho, (15)	Lap. Gyn	Both	50/50	Palonosetron 0.075 mg + Inhaled / Palonosetron 0.075 mg + TIVA	48% / 50% at 24 h, *p > 0.05*
Park and Cho, (16)	Lap. Gyn	Inhaled	45/45	Palonosetron 0.075 mg / ondansetron 8 mg	42.2% / 66.7% at 24 h, *p < 0.05*
Kasagi et al. (17)	Lap. Gyn	TIVA	30/30/30/30	Fentanyl 20 μg.kg^−1^ / fentanyl 20 μg.kg^−1^ + droperidol 2 mg / fentanyl 20 μg.kg^−1^ + naloxone 0.1 mg / fentanyl 20 μg.kg^−1^ + droperidol 2 mg + naloxone 0.1 mg	43% / 43% / 70% / 17% at 24 h, *p < 0.001*
Kawano et al. (18)	Lap. Gyn.	Both	42/42/42	Sevoflurane / propofol 4-8 mg.kg^−1^.h / propofol 2 mg.kg^−1^.h + sevoflurane	62% / 29% / 21% at 24 h, *p < 0.0005*
Soga et al. (19)	Open Gyn.	Inhaled	24/20	Fosaprepitant 150 mg / ondansetron 4 mg	71% / 55% at 24 h, *p > 0.05*
Joo et al. (20)	Lap. Gyn.	Inhaled	50/49/50	IV saline / haloperidol 1 mg / haloperidol 2 mg	42% / 22% / 20% at 24 h, *p = 0.03*
Yang et al. (21)	Lap. Gyn.	Inhaled	50/53/50	Acu+ dexamethasone 10 mg / Tropisetron 5 mg + dexamethasone 10 mg / dexamethasone 10 mg	28% / 26% / 50% at 24 h, *p = 0.048*
Bang et al. (22)	Lap. Gyn.	TIVA	50/50	Palonosetron 0.075 / saline 1.5ml	34% / 58%, *p = 0.027*
Dewinter et al. (23)	Lap. Gyn.	Inhaled	196/196/123	Alizapride 100 mg / Ondansetron 4 mg / Saline 4ml	32.1% / 28.6% / 34.1% in PACU (RR 1.13, 90% CI 0.87–1.45); 36.8%/31.5%/39.3% at 24h (RR 1.17, 90% CI 0.91−1.50)
Geng et al. (24)	Lap. Gyn.	TIVA	65/65	Dexmedetomidine 0.5 μg.kg^−1^ over 10 mins loading, 0.1 μg.kg^−1^.h maintenance / equal volume of saline	5% /14% at 2 h. *p = 0.069*; 38.5% / 43.1% at 24h. *p = 0.592*
Soga et al.Lee (19)	Lap. Gyn.	Inhaled	55/55	Aprepitan 80 mg + ondansetron 4 mg stat + 12 mg into PCA / ondansetron 4 mg stat + 12 mg into PCA	62% / 84% at 24h. *p = 0.011*; 67% / 84% at 48 h. *p = 0.05*.
Lee et al. (25)	Lap. Gyn.	Inhaled	45/44	Ramosetron 0.3 mg EOS + 0.3 mg 4 h postop/ramosetron 0.3 mg EOS + saline 4 h postop	42.2% / 25% at 24h. *p = 0.086*
Kim et al. (26)	Lap. Gyn.	Inhaled	44/44/44/44	Ramosetron 0.3 mg stat + 0.6 mg into PCA / Ramosetron 0.3 mg stat / Palonosetron 0.075 mg / normal saline	8/27/22/33 patients had PONV at 24h; 4/19/17/22 at 48 h; 0/13/14/12 at 72h after discharge from PACU, *p < 0.05*;
Oh et al. (27)	Lap. Gyn.	Inhaled	47/47	Nefopam PCA / fentanyl PCA; rescue ondansetron 4 mg	31.9% / 57.4% at 24 h. *p = 0.022*
Bhakta et al. (28)	Lap. Gyn.	Both	30/30	Propofol + nitrous oxide / Propofol infusion + Isoflurane + nitrous oxide	36.6% / 76.6%, *p < 0.01*
Khan et al. (29)	Lap. Gyn.	Inhaled	70/70	Gabapentin 600 mg /oral placebo 2h. before surgery	32.9% / 64.3% at 24h *p = 0.001*
Seki et al. (30)	Lap. Gyn.	GA / GA + epidural	45/45	12–15 ml 0.5% Ropivacaine + GA / GA with remifentanil infusion + intermittent fentanyl boluses	44.4% / 60% (RR 0.53, 95% CI 0.23–1.23), *p = 0.14*
Omran and Nasr (31)	Mastectomy	Inhaled	40/40	Mirtazapine 30 mg / Ondansetron 16 mg	25% / 35% at 24 h (RR 0.7143, 95 % CI 0.3607–1.414)
Voigt et al. (32)	Elective breast surgery	Both	80/80/80/79 /80/81	Haloperidol 1.25 mg + Tropisetron 2 mg + TIVA / Haloperidol + Tropisetron + Volatile / Dimenhydrinate 31 mg + Dexamethasone 4 mg + TIVA / Dimenhydrinate + Dexamethasone + Volatile / Placebo + TIVA / Placebo + Volatile	25% /17.5% / 15% / 11.4% / 43.8% / 48.1%; halo. + trop. reduced PONV 3.4 x more than placebo (OR 0.30, CI 0.18–0.50, *p > 0.0001*); dimen. + dexa. reduced PONV 5.9 x more than placebo (OR 0.17, CI 0.09–0.30, *p < 0.0001*)
Olanders et al. (33)	Partial mastectomy	Inhaled	37/38	Betamethasone 8 mg / control	57% / 68%, *p = 0.27*

*N, number of patients; PONV, postoperative nausea and vomiting; Drop, droperidol; Trop, tropisetron; Ond, ondansetron; TIVA, total intravenous anesthesia; IV, intravenous; Acu, acustimulation; PACU, post-anesthesia care unit; RR, relative risk; CI, confidence interval; PCA, patient controlled analgesia; EOS, end of surgery; Postop, postoperatively; GA, general anesthesia; TIVA, Total Intravenous Anesthesia; Halo, haloperidol; Dimen, dimenhydrinate; Dexa, dexamethasone*.

## Discussion

### Incidence of PONV in Patients Undergoing Breast and Gynecological Surgery

There is sufficient documentation showing that women undergoing breast and gynecological surgery have a reported incidence of postoperative nausea and vomiting up to 80% to 95% within the first 24 h after surgery when they received insufficient or no prophylactic antiemetic therapy (34–36). Conversely, the occurrence of PONV in this subset of surgical patients can dramatically decrease after the systematic implementations of PONV guidelines (37).

Breast cancer surgery constitutes an additional risk factor for PONV in female surgical patients with a reported incidence of up to 30% to 68% within the first 24 h postoperatively in patients that received intraoperative prophylactic antiemetics (38–40), whereas in non-treated patients PONV frequency increases to 70%-80% of patients (41–43).

Gynecological surgery involves patients who are at high risk for PONV is associated with a higher incidence of PONV (female sex, non-smoking status, and requirement for postoperative opioids) (34). The incidence of PONV in the obstetric and gynecological surgical patients has ranged between 40–80%, especially in laparoscopic surgery (28, 44–46).

### Specific Risk Factors for Postoperative Nausea and Vomiting in Female Surgical Populations

The multifactorial etiology of PONV has been widely studied with the subsequent identification of several independent predictors such as emetogenic factors (e.g., perioperative use of opioids, inhaled or balanced anesthesia, length of anesthesia) and patient-related risk factors (e.g., smoking status, female gender) (2). Being a female patient is the strongest predictor of PONV, followed by the antecedent of episodes of PONV and motion sickness (2, 3, 5). Other known PONV predictors in women are preoperative history of nausea and vomiting during pregnancy, female neonate, and premenstrual syndrome (2, 4). However, it is very important for the anesthesia providers to recognize the presence of other lesser known independent risk factors that enhance the frequency of PONV such as sex hormones levels, psychosocial factors, pharmacogenomic pleomorphism, and ethnicity.

### Hormonal Status According to the Menstrual Cycle

Anecdotally, the incidence trend of emetic episodes increases after menarche and decreases through the menopausal transition (10, 47). Moreover, increased estrogen and progesterone levels during pregnancy have been associated with a prolonged gastrointestinal transit time and a reduction in the esophageal sphincter pressure (10). These facts suggest that cyclic variations in reproductive hormones in females may influence their susceptibility to nausea and motion sickness and therefore, to PONV (4, 6). Previous reports in women revealed that their hormonal status could play an important role in the occurrence of PONV within the first 5 days of the menstrual cycle (48, 49). Based on these assertions, a female patient undergoing major surgery under balanced or inhaled anesthesia, in which postoperative opioid use is expected (e.g., breast cancer surgery or laparoscopic gynecological surgery), is considered at high risk of PONV regardless of her age, smoking status or history of PONV and a multimodal prophylactic approach for PONV (>2 interventions) is highly recommended (5).

The correlation between the menstrual cycle phases and the frequency of PONV has been assessed by several authors, however, there is no firm evidence linking any specific phase of the cycle with a higher propensity for PONV. Nevertheless, an increased incidence of early PONV in women in the follicular and ovulatory stage, when levels of estrogen (estradiol) are higher, compared to those who were in the luteal phase has been reported by several studies (8, 9). Other researchers found a significant association between the ovulatory phase of the menstrual cycle and a higher incidence of early and late PONV when compared to the follicular and luteal phases. In addition, in the study of Zou et al., after multivariate logistic regression analysis showed that the phase of the menstrual cycle was an independent risk factor for early and late PONV (50). Conversely, other studies have concluded that changes in female hormones during the different stages of the menstrual cycle were not associated with an increased incidence of PONV (7, 51). The higher incidence of PONV in premenopausal women has been associated to high plasma levels of estrogen hormones, and to greater requirements of opioids (52, 53). The study conducted by *Kudach et al*. showed an equivalent rate of PONV in female patients up to ≥ 70 years, when the incidence of PONV was significantly lower (52). Therefore, we should consider these variables in female patients undergoing major surgery when assessing the PONV risk factors as described in the current consensus guidelines (4).

### PONV Associated With Tumor Receptor Status in Breast Cancer Surgery

Estrogen and progesterone receptors in the breast tissue are affected by the level of sex hormones and are actively involved in the development of breast cancer; with the endogenous estrogen and progesterone binding specifically to estrogen receptors (ER) or progesterone-receptor (PR), and influencing tumor growth (54). In addition, elevated estrogen levels are also known to increase emesis, suggesting a potential interaction of the estrogen receptor (49). The higher incidence of PONV in premenopausal patients has been linked to elevated estrogen levels (estrone, estradiol, and dehydroepiandrosterone), hence, the higher frequency of PONV observed in postmenopausal women (>50 years) and positive-ER breast cancer also correlates with high estrogen levels (55) (Table 2).

**Table 2:** Physiologic changes associated with an increased risk of postoperative nausea and vomiting in the female population (Independent risk factors).

Preoperative history of severe nausea and vomiting during pregnancy, female neonate, premenstrual syndrome (2, 4)
Follicular and proliferative phase of menstrual cycle (7, 8, 9, 11, 30, 31, 32, 33, 34).
Age ≥ 50 years, previous chemotherapy, and estrogen-positive breast tumor (30, 35, 36).
Preoperative anxiety and stress (36, 38, 39, 40, 41).
Pharmacogenomic pleomorphism (28, 41, 42, 43, 44, 45, 46, 47, 48, 49, 50, 51, 52).
Ethnicity (lower incidence in Black-Africans) (53, 54, 55).

### Preoperative Psychosocial Factors Affecting Women Undergoing Breast and Gynecological Surgery

Preoperative psychological factors such as anxiety and distress may be associated with increased severity of PONV in women with breast cancer (56). Even conservative minor procedures, such as excisional breast biopsy and conservative lumpectomy can be very stressful for women. The onset of preoperative stress in these patients was associated to a variety of factors such as exposure to surgery and anesthesia, experiencing postoperative pain, appearance, scarring, and cancer diagnosis and prognosis (57). Response expectancies based on previous personal or vicarious experiences, have shown to determine immediate postoperative outcomes regarding pain, PONV and fatigue (56, 58–60). In addition, *Montgomery et al*. reported that anxiety and stress, as part of response expectancies, may have an important influence on late post-discharge nausea and vomiting occurrence (59).

### Genomic Pleomorphisms and Ethnicity

Recent studies have demonstrat that previous history of chemotherapy-induced nausea and vomiting (CINV) may contribute to increase the risk of PONV (61). Conversely, there is also evidence showing that patients who have not presented PONV after general anesthesia do not experience CINV either because of different mechanisms including genetic predisposition (47, 61–65).

For instance, polymorphisms in the serotonin transport genes are associated with increased PONV in women with breast cancer, even before receiving chemotherapy (47), while there is a tendency for individuals categorized as CYP2D6 poor metabolizers to experience PONV (66). Moreover, polymorphisms in the serotonin receptor genes HTR3A and DRD3 are linked to a decrease rate of PONV, while on the contrary, HTR3B receptor gene polymorphism may contribute to an increase PONV (67–69). Therefore, pharmacogenomic variability in serotonin transport genes may explain the erratic incidence of PONV and the irregular response to antiemetic medication observed in around 30% of patients undergoing breast cancer surgery (69). Individual carriers of alleles to COMT, DRD3 and TPH genes show a tendency to low PONV frequency (69). Women presenting some genotypes such as Val/Val may experience higher pain intensity, and opioid requirements contributing to increase the occurrence of PONV (especially nausea), when compared with patients with heterozygous V/Met polymorphism (69). The Met/Met genotype has been related with an elevated density of mu receptors, which may explain the reduced levels of pain and opioid consumption observed in those patients (70, 71).

Several studies have demonstrated that ethnicity can be an independent risk factor for PONV, whose incidence shows variations in different ethnic groups, which have so far been more noticeable in Black patients. The effect of ethnicity on PONV could be influenced by pharmacogenomic and cultural factors (72–74). However, although more studies are lacking in various ethnic groups, the existing evidence would raise a question about the validity of the scoring systems derived predominantly from the ethnically Caucasian population and if ethnicity could be used to improve the predictability of PONV in the female surgical population.

## Pharmacological Interventions for Postoperative Nausea and Vomiting in Female Surgical Populations

Postoperative nausea and vomiting persist as one of the commonest complications even though the use of many aggressive antiemetic prophylactic strategies has increased over the last twenty years (75). The growing implementation of the Enhance Recovery After Surgery (ERAS) protocols in most surgical procedures have allowed to tailor the pharmacologic treatment to the patient's risk level of PONV determined by the currently validated risk-scoring system and treatment guidelines (2, 4, 41).

Regarding the pharmacological management of PONV/PDNV, dexamethasone and 5-hydroxytryptamine-3 (5-HT_3_) receptor antagonists are the most common PONV prophylactic medications used among trials. Other pharmacological interventions can be used such as dopamine receptors antagonists, neurokinin-1 (NK-1) receptor antagonists, total intravenous anesthesia (TIVA), gabapentin, nefopam, midazolam, intranasal nicotine, and naloxone were also reported (4, 76). Moreover, there is limited data on non-pharmacological interventions such as the use of transcutaneous acupoint stimulation in female surgical populations (4).

### Dexamethasone

The prophylactic effect of dexamethasone on PONV may vary based on dose administered and population-specific risk. Dexamethasone has proven its effectiveness at dosage of 4–12 mg IV usually combined with other antiemetics (13, 17, 20, 21, 32, 77). *D'Souza et al*. reported a significant reduction in PONV incidence at 3 and 24 h after the prophylactic administration of intravenous (IV) dexamethasone (4 mg) in comparison with IV ondansetron (4 mg) in patients undergoing laparoscopic gynecological surgery under inhaled anesthesia (22.6% vs. 51.6%, *p* = 0.03 and 29% vs. 61%, respectively; *p* = 0.02). Of note, authors excluded patients with past medical history of motion sickness from this trial (13). However, a higher dexamethasone dose (8 mg) was not associated with a significant reduction on PONV occurrence, being this outcome consistent with other published reports in similar surgical populations (13, 17, 20, 21).

In an interesting design, *Kasagi et al*. reported an important reduction in PONV incidence with the combination of droperidol, dexamethasone and naloxone in patients undergoing laparoscopic gynecological surgery under total intravenous anesthesia (TIVA) and who received patient-controlled analgesia (PCA) with fentanyl IV for the management of postoperative pain. Based on Apfel's score (34), more than a half of the patients included in this trial were at high risk of PONV. Then patients were randomly assigned to either one of four groups: droperidol (Dr), dexamethasone (Dx), naloxone (Nx) and combined therapy (Cm). There was a significant reduction in PONV occurrence in the group treated with combined therapy (Cm) (17).

A combination of prophylactic IV dexamethasone and IV haloperidol is also associated with a significant reduction of PONV incidence when compared to placebo in patients at high risk undergoing laparoscopic gynecological surgery under inhaled anesthesia (*p* = 0.003) (25). Likewise, Voigt *et al*. reported a 5.9 times reduction of PONV risk in patients undergoing elective breast surgery who received a prophylactic combination of dimenhydrinate and dexamethasone when compared to a control group (OR 0.17, CI 0.09–0.30; *p* < 0.0001) (32). Other dexamethasone combinations such as dexamethasone + IV tropisetron and dexamethasone + acupoint stimulation have been also associated with a significant reduction in PONV occurrence when compared to dexamethasone alone (*p* = 0.021) (21).

### 5-Hydroxytryptamine-3 (5-HT_3_) Receptor Antagonists

5-HT3 receptor antagonists have proved its effectiveness in PONV/PDNV prophylaxis and are the most recommended first-line prophylactic treatment for PONV (4, 5, 76, 78, 79). Recent clinical trials showed the efficacy of newer 5-HT3 receptor antagonist in reducing the incidence of PONV in gynecological and breast surgery (15, 16, 22, 80–82). In a prospective controlled trial comparing the effect of prophylactic palonosetron on PONV after gynecological laparoscopic procedures, *Bang et al*. reported a substantial reduction on PONV occurrence when compared to placebo (22). Moreover, *Park et al*. compared the PONV prophylactic effect of IV palonosetron (0.075 mg) with IV Ondansetron (8 mg) in patients with Apfel's score ≥2 finding a significant decrease in PONV incidence at 24 h in the palonosetron group when compared to ondansetron (42.2% vs. 66.7%, respectively; *p* < 0.05), although there were no significant differences between groups within the first 6 postoperative hours (15). The longer half-life of palonosetron compared to ondansetron could have influenced these outcomes (83). To our knowledge, no studies have been published describing the PONV incidence after postoperative day 1 in female patients receiving palonosetron or assessing cost-benefit of palonosetron administration on surgical patients at high-risk of PONV. Additionally, the effect of a prophylactic dose of palonosetron on the incidence of PONV is comparable to the results obtained with the administration of TIVA in this patient setting (16). In a recent report, *Hong et al*. compared the effectiveness of palonosetron (P) with the combination of midazolam-palonosetron (MP) in 104 patients undergoing breast cancer surgery. From 0 to 24 h after surgery with no intergroup statistical significance (42.3% and 48.1%) (81).

Ramosetron was also compared to palonosetron in female patients undergoing gynecological laparoscopic surgery in a study conducted by *Kim et al*. (26). They reported no significant differences on PONV occurrence in patients receiving one prophylactic IV dose of ramosetron (0.3 mg) when compared to 2 doses, one prophylactic and another dose 4 h after gynecological laparoscopic surgery (80).

### Neurokinin-1 (NK-1) Receptor Antagonists

Aprepitant and fosaprepitant are the NK-1 receptor antagonists with long elimination half-life most studied as preventive treatment for PONV (84). An early study carried out by *Gesztesi et al*. revealed that NK-receptor antagonist CP-122,721 (200 mg PO), was more effective than ondansetron lowering the frequency of PONV in the first 24 following gynecological surgery (85). In a prospective study, *Soga et al*. compared the efficacy of fosaprepitant (150 mg IV) to ondansetron (4 mg) in 44 patients undergoing gynecological laparoscopic surgery under balanced general anesthesia and receiving epidural fentanyl in PCA pump for postoperative pain management. Even though complete response was similar between groups, there were no vomiting episodes reported in patients receiving fosaprepitant, whereas 4 patients experienced vomiting in the ondansetron group (0% vs. 20% respectively, *p* < 0.05) (19).

Moreover, the efficacy of oral aprepitant combined with IV ondansetron compared with ondansetron alone for PONV prophylaxis was studied by *Ham et al*. in patients with ≥2 risk factors for PONV and undergoing laparoscopic gynecological surgery (86). The occurrence of PONV at 24 h was significantly lower in the aprepitant +ondansetron group when compared to ondansetron group (62% vs. 84%, respectively; *p* = 0.011). However, this difference was not maintained at 48 h.

### Dopamine Receptor Antagonists

Dopamine receptor antagonists (e.g., butyrophenones) have successfully been used for prevention and treatment of PONV in female surgical populations. However, effective doses are commonly linked to side effects such as sedation and extrapyramidal symptoms, hence limiting their perioperative use. Joo *et al*. randomized 150 female patients considered at high-risk of PONV and undergoing gynecological laparoscopic surgery into 3 groups: normal saline (Group H0), haloperidol 1 mg (H1), or haloperidol 2 mg (H2). The authors reported a significant reduction in PONV occurrence in both haloperidol groups when compared to normal saline (H1 = 29%, H2 = 24% and H0 = 54%, *p* = 0.001), although higher levels of sedation occurred in patients receiving 2 mg of haloperidol (H2 group) (20). *Ekinci et al*. compared the incidence of severe PONV in patients undergoing gynecologic procedures and receiving different prophylactic medications such as droperidol (2.5 mg), metoclopramide (10 mg), tropisetron (2.5 mg), ondansetron (4 mg), or saline (control group). The overall PONV incidence was 48%, being the lowest incidence of severe emesis observed in the ondansetron group compared to droperidol, metoclopramide, tropisetron, and saline (15%, 20%, 40%, 25%, and 60% respectively) (14). This finding correlates with previous reports describing the lack of efficacy of low metoclopramide doses for PONV prophylaxis (5).

To our knowledge, only one study has described the PONV incidence in patients receiving alizapride, another dopamine antagonist commonly used in oncology. *Dewinter et al*. found no significant differences in PONV occurrence after the administration of alizapride in patients at high risk of PONV undergoing laparoscopic gynecological surgery when compared to ondansetron (23).

### Other Pharmacological Interventions

The impact of a single prophylactic dose of betamethasone on PONV in patients undergoing breast surgery was assessed by *Olanders et al*. Patients were randomized to receive IV betamethasone or no prophylaxis before surgery. The authors reported no significant intergroup differences in the overall PONV incidence. Nevertheless, severity of nausea between postoperative hours 4–12 was significantly lower in the group of patients receiving betamethasone (23% vs. 50%, *p* < 0.05) (33).

Considering that preoperative anxiety may play an important role in the onset of PONV, *Omran et al*. compared the PONV prophylactic effect of oral mirtazapine (30 mg), an antidepressant, to oral ondansetron (16 mg) in 80 patients undergoing mastectomy. Even though patients in the mirtazapine group experienced significantly lower preoperative anxiety levels, no differences were found in overall PONV incidence between groups (31).

In contrast, the short-acting benzodiazepine midazolam may be effective in diminishing PONV, especially when used combined with other antiemetics or as part of a multimodal antiemetic therapy in breast and other cancer-related surgeries (81, 82, 87, 88). A meta-analysis conducted by Grant *et al*. determined that midazolam was associated with a significant decrease in overall PONV rates as well as when used as rescue antiemetic medication (89). Similarly, *Ahn et al*. reported that patients medicated with midazolam presented lower incidence of PON, POV, and PONV (RR, 0.45; 95% CI, 0.36–0.57; I^2^ = 31%; NNT = 3; *n* = 7) (90). Although the exact mechanism for the antiemetic action of midazolam remains unknown, it has been proposed that midazolam may decrease dopamine synthesis and release by direct action on the chemoreceptor zone or by blocking adenosine reuptake (91). Although anxiolysis may contribute to the antiemetic effects of midazolam, binding to the Gamma-Aminobutyric Acid (GABA)-benzodiazepine complex reduces 5-HT_3_ release and dopaminergic neuronal activity (18, 92, 93).

*Kamali et al*. conducted a double blind randomized clinical trial to compare the effectiveness of ginger 1 mg (before and after anesthesia) with dexmedetomidine 25 mg (before surgery) in preventing PONV after hysterectomy (94). The group of patients treated with ginger showed a lower incidence of nausea (*p* = 0.02) and vomiting (*p* = 0.03) than the dexmedetomidine group within the first 2 h postoperatively. Beyond this timepoint, there were no differences between groups (94).

## Non-Pharmacological Interventions for Postoperative Nausea and Vomiting in Female Surgical Populations

There are reports describing the use of acupoint electrical stimulation to reduce the incidence of PONV after breast surgery. However, its efficacy remains controversial (95–98). *Küçük et al*. studied the effect of acupressure on PONV occurrence after gynecological surgery (99). Patients were randomly allocated into three groups: to acupoint point P6 (both wrists) 1 h before surgery, to K-K9 point (both hands) 30 min before the end of surgery, and a control group (routine care). At 24 hours after surgery, patients in the K-K9 group experienced less nausea than the control group (*p* < 0.05), and less retching than patients in the P6 group (*p* < 0.05*)* (99). The results of a recent meta-analysis conducted by *Sun et al*. showed that non-needle acupoints stimulation also reduced the incidence of PONV in patients at moderate risk of PONV. However, the low quality and limited number of studies included in this meta-analysis did not allow for definite conclusions and recommendations (97).

## Anesthetic Management and Postoperative Nausea and Vomiting in Female Surgical Populations

Even though female gender, non-smoking status, past medical history of PONV (or motion sickness) and postoperative use of opioids are recognized as the main risk factors for PONV (34), other secondary variables (e.g., age <50 years, gynecological surgery, laparoscopic surgery) should be considered when determining the overall individual PONV risk (5).

There is a weak association between intraoperative use of opioids and PONV occurrence. However, inhaled anesthesia (i.e., volatile anesthetics and/or nitrous oxide) is considered the main predictor of PONV related to the anesthetic management (level of evidence A1) (5). Inhaled general anesthesia is associated with increased incidence of early PONV (0–2 h after surgery) but has no impact in delayed PONV (2, 100).

Propofol infusion is widely known to improve PONV outcomes in female surgical patients when compared to balanced anesthesia (18, 28). *Kawano et al*. studied the incidence of PONV at 0–2 h and 0–24 h after gynecological laparoscopic surgery in 126 women. Patients were randomly assigned to receive general anesthesia with either sevoflurane (Group S), propofol (Group P), or a combination of propofol and sevoflurane (Group PS) (18). Immediately after surgery (0–2 h) and up to 24 h a significantly greater number of patients in the P and PS groups experienced a complete response when compared to group S (*p* = 0.001 and *p* < 0.0005 respectively). Nausea was also more frequent in the Group S than in the other two groups (Group S = 62%, Group P = 29% and Group PS = 21%; *p* < 0.0005) (18). Likewise, *Bhakta et al*. reported a significant reduction in postoperative emesis with the use of propofol infusion when compared to isoflurane anesthesia in patients undergoing gynecological laparoscopic surgery (28).

The use of inhalation anesthetic agents is associated with a dose-dependent rise in PONV prevalence (2, 100). In a retrospective study, *Morita et al*. reviewed 928 patients undergoing breast cancer surgery under inhalation anesthesia (101). Their results showed that the use of desflurane and the duration of anesthesia were independent risks factors for early PONV, whereas Apfel score and duration of anesthesia were considered by the authors independent risks factors for delayed PONV (101).

Dexmedetomidine as part of a TIVA approach or in combination with dexamethasone may improve PONV outcomes in female surgical populations (24, 102). In a randomized, controlled, double-blind trial, *Kwak et al*. demonstrated the efficacy of dexmedetomidine alone and in combination with dexamethasone to prevent PONV when compared to placebo after breast surgery (102). The incidence of PONV was significantly higher in the placebo group compared with the dexmedetomidine group and the dual group during both, at PACU stay (12%, 4%, and 3%, respectively) and within the first 24 h after surgery (70%, 20% and 12%, respectively). They concluded that dexmedetomidine alone or in combination with dexamethasone was equally effective in decreasing the occurrence of PONV in this subset of patients (102).

The antiemetic effect of dexmedetomidine may be mediated by a modulatory action on the post-synaptic α_2A_ receptors acting as heteroreceptors, and reducing the release of 5-HT in the dorsal and median raphe nucleus located in cerebellum and mid-brain pons respectively (103). Other proposed antiemetic mechanisms of dexmedetomidine are the modulatory effect on dopamine release in the nucleus recumbens (104) and the suppression of histamine-mediated production of pro-inflammatory interleukine-6 (IL-6) (105).

The incidence of PONV was studied in patients undergoing laparoscopic gynecological surgery under an opioid-sparing anesthesia technique by *Seki et al*. They randomly assigned 90 patients to receive either general anesthesia alone (group G) or a combination of general anesthesia and epidural block with ropivacaine (group GE). All patients received PONV prophylaxis with dexamethasone and anesthesia maintenance with sevoflurane. Even though patients in the group G received more intra- and postoperative opioids, the authors found no significant difference when comparing PONV incidence among groups (RR:0.53, 95% CI: 0.23–1.23, *p* = 0.14) (30).

Other opioid-sparing analgesic approaches are the administration of nefopam, a centrally acting analgesic mostly used for neuropathic pain management, and gabapentin. *Chung-Sik Oh et al*. randomized 94 patients to receive either nefopam- or fentanyl-based PCA for pain management after gynecological laparoscopic surgery under total intravenous anesthesia (TIVA). The use of nefopam was associated with a significant decrease in PONV occurrence when compared to fentanyl (31.9% vs. 57.4% respectively, *p* = 0.022) (27). Likewise, *Khan et al*. reported a significant decreased PONV incidence after oral gabapentin (600 mg) compared to placebo in patients undergoing diagnostic gynecological laparoscopy surgery (32.9% vs. 64.3% respectively, *p* < 0.001) (29).

The development of Enhance Recovery After Surgery (ERAS) pathways for different surgical specialties, including cancer breast surgery, has led to a reduction in the prevalence of PONV, although the number of studies remains limited (106, 107). The growing use of multimodal perioperative analgesia strategies in ERAS protocols contributes to an effective management of postoperative pain with a considerable reduction in the amount of perioperative opioid use through the combination of non-opioid pharmacological management and regional anesthesia techniques, which consequently, decreased the prevalence of PONV (106, 108, 109). A recent retrospective study by *Chiu et al*. clearly showed a drastic reduction in PONV occurrence after the initiation of ERAS pathways for total mastectomy compared with a non-ERAS cohort (28% vs. 50%, respectively; p<0.001*)* (107). The use of regional nerve blocks (e.g., pectoral blocks or PECS, paravertebral, erector spinae plane block, and interfascial plane block) as a central component of multimodal opioid-free perioperative analgesia has had a significant impact on the frequency of PONV after breast surgery (110–114).

## Conclusions and Future Directions

Despite the efforts made by health care providers and researchers to reduce the occurrence of PONV in female patients at high risk, breast and gynecological surgery constitute additional risks factors for PONV, with an incidence that reaches 30–68% in the first 24 postoperative hours even in patients who have received prophylactic antiemetic treatment. Even though published data is limited, other variables such as sex hormone levels (especially estrogens) in pre and post-menopausal women, preoperative psychosocial status, pharmacogenomic pleomorphisms, and ethnicity, which can be considered independent risk factors must be considered when assessing the risk of PONV in female surgical populations.

Overall risk stratification and increasing compliance with the consensus for PONV management may positively influence clinical outcomes. While novel drugs are continuously under research, future randomized clinical trials should aim to identify both pharmacological and non-pharmacological alternatives that could potentially decrease the current threshold of PONV incidence in female surgical patients.

## Author Contributions

ME-V performed literature search, worked on the structural design and methodology, and wrote and authored the manuscript. JF-D and AU collaborated equally in developing the methodology, collecting the data, and writing the manuscript. SB provided the publication concept, editorial advice, and supervised the production. All authors reviewed the final manuscript before submission. All authors contributed to the article and approved the submitted version.

## Conflict of Interest

The authors declare that the research was conducted in the absence of any commercial or financial relationships that could be construed as a potential conflict of interest.

## Publisher's Note

All claims expressed in this article are solely those of the authors and do not necessarily represent those of their affiliated organizations, or those of the publisher, the editors and the reviewers. Any product that may be evaluated in this article, or claim that may be made by its manufacturer, is not guaranteed or endorsed by the publisher.

## Abbreviations

ERAS: Enhance Recovery After Surgery
GA: general anesthesia
NIH: National Institutes of Health
PONV: postoperative nausea and vomiting
PON: postoperative nausea
POV: postoperative vomiting
TIVA: Total Intravenous Anesthesia.
